# Short-term ambient heat exposure and low APGAR score in newborns: A time-stratified case-crossover analysis in São Paulo state, Brazil (2013–2019)

**DOI:** 10.1371/journal.pgph.0004557

**Published:** 2025-09-05

**Authors:** Michelle Del Carretto, Audrey Godin, Danielson Neves, Enny S. Paixão, Kai Wan, Julia Pescarini, Andrêa Ferreira, Taísa R. Cortes, Liam Smeeth, Maurício L. Barreto, Elizabeth B. Brickley, Chérie Part

**Affiliations:** 1 Faculty of Epidemiology and Population Health, London School of Hygiene & Tropical Medicine, London, United Kingdom; 2 Centro de Integração de Dados e Conhecimentos para Saúde, Fundação Oswaldo Cruz, Salvador, Brasil; 3 Faculty of Public Health and Policy, London School of Hygiene & Tropical Medicine, London, United Kingdom; Federal University of Bahia: Universidade Federal da Bahia, BRAZIL

## Abstract

Exposure to high ambient temperatures near the time of delivery has been associated with adverse birth outcomes, but studies examining the impact on immediate newborn health remain limited. We used a time-stratified case-crossover design combined with a distributed lag nonlinear model to evaluate the short-term effects of ambient heat (0–1 day lag) on low 5-minute APGAR score (≤7; sub-categories: 6–7, 3–5, 0–2). Cases of low APGAR score among low-risk births (*n* = 34,980) in São Paulo state (274 municipalities), 2013–2019, were extracted from Brazil’s Live Birth Information System (*Sistema de Informações Sobre Nascidos Vivos*). Municipality-level daily mean temperatures were constructed from ERA5-Land reanalysis data and linked with case and control days by date and municipality of delivery. Models were adjusted for relative humidity and stratified by maternal age, race/ethnicity, education, parity, timing of prenatal care initiation, infant sex, municipality-level deprivation, and Köppen climate zone. Overall, exposure to high (95^th^ percentile: 26.1°C) versus moderate (50^th^ percentile: 20.9°C) temperature 0–1 days before delivery was associated with 8% higher odds (OR: 1.08, 95% CI: 1.02-1.14) of low APGAR score (≤7). In stratified analyses, heat-associated risks were elevated among infants born to women with <12 years of schooling (1.10, 1.03-1.17) and/or self-identifying as Brown/*Parda* (1.10, 1.01-1.20). Associations were primarily driven by same-day (lag 0) exposure and were only observed in newborns with moderately low APGAR scores (6–7). Acute exposure to ambient heat may adversely impact newborns’ immediate health in low-risk live-births, highlighting the need for heat mitigation measures near the time of delivery.

## Introduction

Rising global temperatures pose a substantial threat to maternal and newborn health [1]. Exposure to ambient heat during pregnancy increases the risk of maternal complications [2] and adverse birth outcomes, such as preterm birth, stillbirth, and low birth weight [3]. Studies focusing on neonatal outcomes remain scarce. There is evidence for an increased risk of neonatal hospital admissions during heatwaves in Brazil and India [4,5], however these studies have limited ability to determine critical windows of susceptibility. Neonatal intensive care admissions include in-hospital transfers immediately post-birth as well as admissions up to 28 days of life. Even when examining the effects of same-day exposure among term births, studies have yet to determine the stage at which exposure to ambient heat poses the greatest risk [4].

The APGAR score is used worldwide to measure newborn health and response to resuscitation interventions at 1-, 5- and 10-minutes of life [6]. A low score indicates poor immediate health condition, and has been associated with long-term health concerns, such as cerebral palsy and epilepsy [7]. Prematurity and low birth weight are risk factors for low APGAR scores. However, even among term, optimal birth weight newborns, this measure is particularly sensitive to characteristics of the labour and delivery, including prolonged labour and surgical (caesarean) birth [8,9].

**Fig 1:**
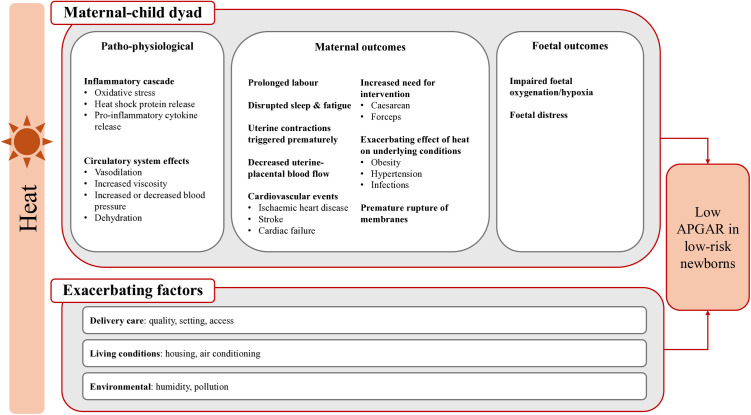
Hypothesised mechanisms through which short-term ambient heat exposure increases the risk of low APGAR-5’ scores.

There is some evidence that low APGAR scores are associated with exposure to high ambient temperatures during the third trimester of pregnancy [10–14]. However, previous studies often averaged temperature exposures over broad windows of susceptibility, such as months or trimesters. We hypothesise that acute exposure to ambient heat, during labour and delivery, influences newborns’ immediate health through several interconnected mechanisms (Fig 1). Circulatory system changes (e.g., dehydration, vasodilation) can reduce uterine-placental blood flow, hindering blood-oxygen exchange with the foetus [15] or triggering maternal cardiovascular events [16]. Inflammatory pathways may cause Premature Rupture of Membranes when combined with prior infection [17]. Discomfort and heat stress may cause fatigue and prolonged labour [18]. These pathways may also increase the need for surgical intervention during delivery, contributing to foetal distress [19]. Heat effects on maternal and newborn health may also interact with aspects of delivery care (e.g., healthcare workforce strain and quality of care), living conditions (e.g., limited access to air conditioning) and environment (e.g., air pollution), all of which are inherently interlinked with the social determinants of health [20,21].

Using a time-stratified case-crossover design, we evaluated the short-term association between daily mean temperature and the odds of low APGAR score at 5-minutes of life (APGAR-5’) in São Paulo state, Brazil. Cases were restricted to low-risk live-births (defined here as singleton, term births with optimal birth weight, cephalic presentation, and absence of congenital anomalies) to remove competing biological pathways. São Paulo state is situated in the Southeast region of Brazil, where the last decade has seen rising temperatures and more frequent, intense heatwaves [22]. Given the unequal burden of heat-health outcomes according to education level and race/ethnicity in Brazil [23,24], subgroup analyses of socio-economic relevance were conducted in addition to those of clinical significance to identify high-risk groups for targeted intervention.

## Methods

### Study setting

São Paulo state is highly urbanised, with a total population of more than 44 million [25]. As defined by Köppen climate designations, the state is characterised by temperate (C) and tropical (A) climates, with a majority of the population living in humid-subtropical, oceanic areas (Cfa, Cfb - hot, humid or mild summers and mild winters, S1 Fig) [26]. Extremely hot summers of 2013/14 and 2014/15 highlight the state’s vulnerability to climate change [22]. Approximately 99% of births in Brazil occur in hospitals [27]. Despite Brazil’s universal healthcare system providing free, essential maternity services, São Paulo state suffers from inequalities in access for certain ethno-racial and socio-economic groups [28,29].

### Ethics statement

As the study analyzed de-identified publicly available data, ethical approval was not required in accordance with Resolução N° 510 (7 April 2016) of the Brazilian Ethics System (Sistema CEP-CONEP) and confirmed with the London School of Hygiene and Tropical Medicine Research Ethics Committee (Reference 30472 and 30657).

### Data

#### Birth data and outcome assessment.

De-identified, individual-level data on live births were downloaded from the Brazilian Ministry of Health’s Unified Health System data registry (*Departamento de Informática do Sistema Único de Saúde*, DATASUS) on 28 May 2024 using the Live Births Information System (*Sistema de Informações Sobre Nascidos Vivos*, SINASC). SINASC contains information on the pregnant person (e.g., age, municipality of delivery), the pregnancy (e.g., completed weeks’ gestation) and the newborn (e.g., APGAR scores, birth weight).

Between 01 January 2013 and 31 December 2019, 4,166,174 live, singleton births were registered in São Paulo state. Births occurring outside the state (N = 4,575; 0.11%) and those with missing 5-minute APGAR score (N = 19,404; 0.47%) were removed. Data were restricted to newborns from low-risk births by excluding infants with: (i) birth weight <2500 or >4000 grams (N = 499,493; 11.99%), (ii) gestational age < 37 or >41 weeks (N = 482,706; 11.59%), (v) congenital anomalies (N = 51,120; 1.23%), (vi) non-cephalic presentation (N = 145,044; 3.48%), as well as births with missing data on these variables (N = 96,117; 2.31%) (S2 Fig). The final dataset included 3,168,273 low-risk births.

The APGAR score is a routine, standardized index conducted by a birth attendant at 1- and 5-minutes after birth. Newborns are scored 0–2 on five criteria (colour/peripheral cyanosis, heart rate, response to stimulation, muscle tone, and breathing), yielding a total score between 0 and 10 [6]. Our primary outcome was a low-risk live-birth of a newborn with an APGAR score of 7 and below (≤7) at 5-minutes of life (hereafter, low APGAR-5’). Secondary outcomes were subcategories of low APGAR-5’: 0–2, 3–5 and 6–7, in line with Brazilian reporting standards [27].

#### Exposure assignment.

Hourly 2-metre air and dew point temperatures (downloaded in Kelvin and converted to Celsius) were obtained for São Paulo state, at a 0.1° x 0.1° (~9 x 9 km) spatial resolution, from the Copernicus Climate Data Store as part of the European Centre for Medium-Range Weather Forecasts’ (ECMWF’s) ERA5-Land reanalysis data [30]. Municipality-level daily mean temperatures (°C) were calculated from hourly air temperatures arithmetically averaged by date over grid cells within municipality boundaries. Relative humidity (%) was calculated as the ratio between real vapor pressure and saturation vapor pressure obtained using Tetens equation with 2-meter dewpoint and air temperatures respectively.

Daily mean temperature was considered the best approximation of daily temperature exposures (accounting for both daytime and night-time temperatures). Our window of exposure was the day of delivery (lag 0) and day preceding delivery (lag 1), approximating childbirth and labour. Exposures were linked to outcomes by date and municipality of delivery. Population-weighted percentiles of daily mean temperature were calculated for São Paulo state over the study period (2013–2019). High and low temperatures were defined as the 95th and 5th percentiles of daily mean temperature, respectively.

#### Municipality-level metrics.

Municipality-level deprivation data were obtained from Oswaldo Cruz Foundation’s Centre for Data and Knowledge Integration for Health (*Centro de Integração de Dados e Conhecimentos para Saúde*) [31]. These data reflect the Brazilian Deprivation Index (*Índice Brasileiro de Privação*, IBP); a composite index based on the 2010 census data on low-income households (per capita income ≤50% minimal wage), illiterate individuals aged ≥7 years, and individuals with inadequate access to water and sanitation. We used population-weighted quintiles, based on deprivation at the countrywide level, and ordered from the least (1st quintile) to the most deprived (5th quintile) municipalities (S1 Fig).

The Köppen classification system assigns climatic regions using temperature and precipitation and allows an understanding of regional biomes, weather and local infrastructural adaptation. Köppen designations for each municipality were obtained from Alvares *et al*. [26].

### Statistical analysis

Daily mean temperature and rate of low APGAR-5’ score (N cases/ N low-risk births) in São Paulo state were plotted against day-of-year to highlight seasonal patterns. Scatterplots were used to examine the functional form of the unadjusted relationships between temperature, relative humidity and rate of low APGAR-5’ score at 0- and 1-day lags. The association between proposed effect modifiers and APGAR-5’ scores (≤7 vs ≥ 8) were assessed using Chi-square tests for independence.

We used a bi-directional time-stratified case-crossover design to estimate the short-term association between daily mean temperature and low APGAR-5’ score. This design has been widely used to assess acute impacts of environmental exposures on health, including birth outcomes [32,33]. Cases act as their own controls, whereby exposure on the day of the health event (case day) is compared to exposure on otherwise similar days when the health event did not occur (control days). Control days were selected as the same day-of-week, calendar month and year as the case day, resulting in 3–4 control days per case. By design, this approach controls for day-of-week effects, seasonal and long-term trends, as well as time-fixed (or slow-changing) individual-level confounders (e.g., socio-economic position, age).

Conditional logistic regression was used to compare the likelihood of exposure to high and low temperatures on case days versus matched control days. We applied distributed lag nonlinear models (DLNMs) to capture potential nonlinear effects of temperature on the day of delivery and day prior (0–1 day lag). The temperature dimension was modelled using a natural cubic spline with one internal knot, while the lag dimension was modelled using a linear term. The number and positioning of spline knots were determined through minimisation of Akaike Information Criterion (AIC) and examination of diagnostic plots. Relative humidity (RH) was adjusted for as a time-varying confounder. RH was averaged over the 0–1 day lag and fitted using a spline with 3 degrees of freedom (*df*). The same model terms were used to analyse secondary outcomes. Effect estimates were expressed as the odds ratio (OR) of low APGAR-5’ score at the 95^th^ and 5^th^ percentiles of daily mean temperature, compared with exposure to the 50^th^ percentile. OR of low APGAR-5’ with exposures on 0 and 1 lag days before delivery (2-day cumulative) are presented, with reference to the contribution of individual lags where relevant.

#### Subgroup analysis.

Subgroup analyses were used to assess potential effect modification by individual and area-level characteristics, including maternal age (<20 years, 20–34 years, > 34 years), maternal race/ethnicity (Brown/*Parda*, Black/*Preta*, White/*Branca*, Asian-descent/*Amarela* and Indigenous*/Indígena*), maternal education (<12 years, ≥ 12 years), parity (nulliparous, primiparous, multiparous), timing of prenatal care initiation (in the first trimester of pregnancy, delayed), infant sex (male, female), municipality-level deprivation (in quintiles), and Köppen climate zones. Stratum-specific ORs were estimated using the same model terms as described above, to ensure comparability between groups. ORs for Köppen climate zones were centered at the population-weighted percentiles of daily mean temperature of either tropical or temperate regions.

#### Sensitivity analysis.

As sensitivity analyses, models were respecified with (i) increased flexibility in the exposure-response curve (natural cubic spline with 2 internal knots); (ii) removal of humidity adjustment; and (iii) a different lag structure (lag 0 only; 0–6 day lag, fitted using a natural cubic spline with one internal knot in the lag and temperature dimensions). We also conducted an equivalent case-crossover analysis using conditional quasi-Poisson regression to ensure our findings were robust to adjustment for daily birth counts (rate denominator), overdispersion, and autocorrelation [34]. Finally, to assess the potential selection bias caused by restricting our sample to low-risk births, we repeated the analysis on all singleton births between 2013–2019 in São Paulo state.

All analyses and figures were conducted in R (4.2.3) using Rstudio and facilitated by the following packages: survival [35], dlnm [36], splines [37] and geobr [38]. The study followed the STROBE reporting guidelines.

## Results

Between 2013 and 2019, 34,980 (1.1%) of the 3,168,273 newborns with low-risk births in São Paulo state had a low APGAR-5’ score (≤7). The annual rate peaked at 1.2% in 2015 and subsequently decreased to 1.0% by 2019 (S1 Table). Low scores (≤7) were more common among male newborns and offspring of women who were younger (<20 years), nulliparous, had < 12 years of education, self-identified as Brown/*Parda* or Black/*Preta*, and/or had later prenatal care initiation (Table 1).

**Table 1:** Characteristics of low-risk births (n = 3,168,273) in São Paulo state, Brazil (2013-2019).

Category	APGAR-5’ score 0–7	APGAR-5’ score 8–10	*P*-value*
	N (%)	N (%)	
**Totals**	34,980	3,133,293	
**Maternal Age**			χ^2^ (2) = 454.8, *p* < 0.001
<20 years	5,858 (16.8)	404,187 (12.9)	
20-34 years	23,764 (67.9)	2,222,700 (70.9)	
≥35 years	5,358 (15.3)	506,400 (16.2)	
Missing	0	6	
**Maternal Education**			χ^2^ (1) = 878.2, *p* < 0.001
<12 years	28,687 (82.2)	2,353,730 (75.4)	
≥12 years	6,194 (17.8)	768,931 (24.6)	
Missing	99	10,632	
**Maternal Race/Ethnicity**			χ^2^ (4) = 582.3, *p* < 0.001
White/*Branca*	17,909 (51.6)	1,793,980 (57.7)	
Brown/*Parda*	14,171 (40.8)	1,114,328 (35.8)	
Black/*Preta*	2,451 (7.1)	178,441 (5.7)	
Asian/*Amarela*	152 (0.4)	19,117 (0.6)	
Indigenous/*Indígena*	59 (0.2)	4,607 (0.2)	
Missing	238	22,820	
**Parity**			χ^2^ (2) = 1391, *p* < 0.001
Nulliparous	19,247 (55.8)	1,408,894 (45.8)	
Primiparous	8,935 (25.9)	1,007,129 (32.8)	
Multiparous	6,303 (18.3)	659,598 (21.5)	
Missing	495	57,672	
**Prenatal Care Initiation**			χ^2^ (1) = 54.2, *p* < 0.001
During first trimester	28,490 (84.7)	2,627,835 (86.1)	
After first trimester	5,155 (15.3)	425,084 (13.9)	
Missing	1,335	80,374	
**Newborn Sex**			χ^2^ (1) = 587.7, *p* < 0.001
Male	19,996 (57.2)	1,586,949 (50.7)	
Female	14,984 (42.8)	1,546,344 (49.4)	
Brazilian Deprivation Index (IBP) **			χ^2^ (3) = 149.3, *p* < 0.001 ^
1 (Least deprived)	11,659 (33.3)	1,110,639 (35.5)	
2	17,682 (50.6)	1,580,246 (50.4)	
3	5,196 (14.9)	403,342 (12.9)	
4	443 (1.3)	39,060 (1.3)	
5 (Most deprived)	0 (0.0)	5 (0.0)	
Missing	0	1	
**Köppen Climate Zones**			χ^2^ (5) = 748.0, *p* < 0.001
Af - Tropical Rainforest (no dry season)	2,171 (6.2)	133,837 (4.3)	
Aw - Tropical Savanna (dry winters)	1,482 (4.2)	201,809 (6.4)	
Cfa - Temperate Humid-subtropical (no dry season, hot summer)	6,971 (19.9)	651,614 (20.8)	
Cfb - Temperate Oceanic (no dry season, warm summer)	20,744 (59.3)	1,760,430 (56.2)	
Cwa - Temperate Humid-subtropical (dry winter, hot summer)	3,320 (9.5)	351,980 (11.2)	
Cwb - Temperate Highland-subtropical (dry winter, warm summer)	292 (0.8)	33,622 (1.1)	
Missing	0	1	

*P-values were obtained from the Chi-square test for independence. Missing values were excluded from the calculation.

^ IBP 5 was excluded from the Chi-square test due to small sample size.

** The Brazilian Deprivation Index is based on the country as whole, allowing comparability of municipality-level deprivation across regions of Brazil.

**Fig 2:**
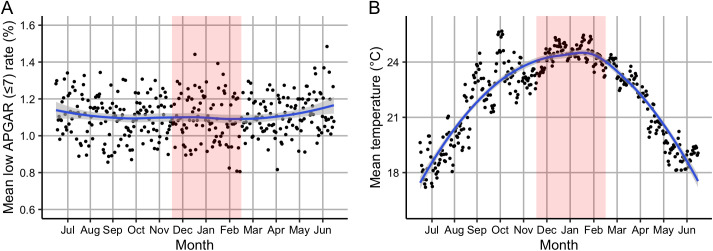
Seasonal patterns in daily rate (%) of low APGAR scores and daily mean temperature (°C). (A) Daily mean rate (%) of low APGAR-5’ scores (≤7) among low-risk deliveries, and (B) daily mean temperature (°C), by day-of-year across all municipalities in São Paulo state (2013–2019). The blue line was fitted using Locally Estimated Scatterplot Smoothing (LOESS) to illustrate seasonal trends. Summer months (December to February) are shaded in red.

Daily rates of low APGAR-5’ scores showed little seasonal variation (Fig 2). Based on population-weighted daily mean temperatures, the high (95^th^ percentile), moderate (50^th^ percentile), and low (5^th^ percentile) temperatures were 26.1°C, 20.9°C, and 14.8°C, respectively.

**Fig 3:**
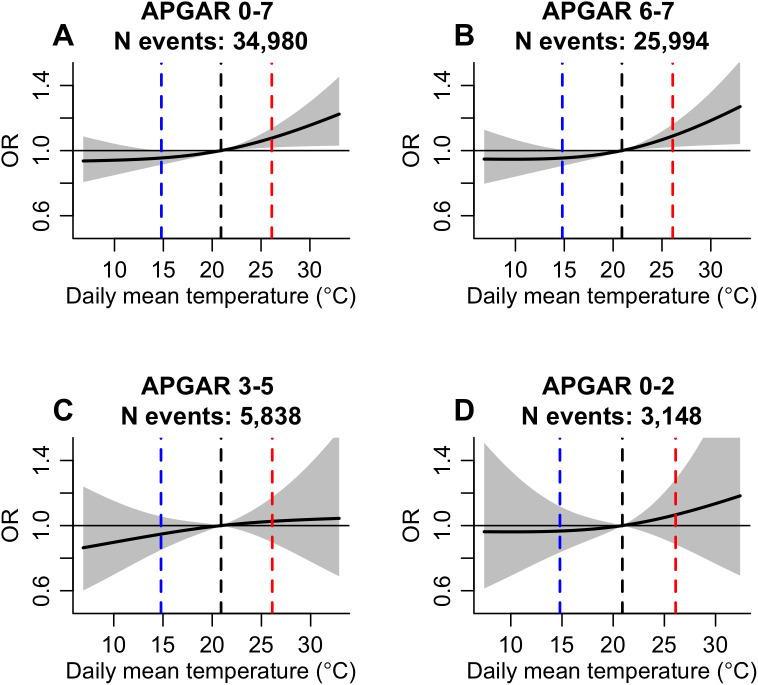
Association between ambient temperature exposures and odds of low-risk delivery with low APGAR-5’ score. Odds ratio (black line) and 95% confidence interval (grey shading) of low APGAR-5’ score (a); 0-7 (b) 6-7; (c) 3-5; and (d) 0-2 with exposure to daily mean temperatures 0-1 days before delivery (2-day cumulative). Predictions were centred at the 50th percentile of population-weighted daily mean temperature (20.9°C; black dashed line). Red dashed lines show the 95th percentile (26.1°C), and blue dashed lines show the 5th percentile (14.8°C) of population-weighted daily mean temperature.

Regression coefficients and fit statistics for models testing the association between daily mean temperature exposures and low APGAR-5’ score subcategories are presented in S2 Table. Odds of low APGAR-5’ score (≤7) increased by 8% (OR: 1.08, 95% CI: 1.02-1.14) with exposure to high versus moderate temperature (26.1°C vs 20.9°C) 0–1 days before delivery (2-day cumulative) (Fig 3A). The effect of high temperature was largely confined to the day of delivery (lag 0; OR: 1.07, 1.00-1.15), with no evidence of association on the preceding day (lag 1; OR: 1.01, 0.94-1.07) (S3 Table). There was no evidence that 2-day cumulative exposure to low temperature (14.8°C vs 20.9°C) was associated with low APGAR-5’ scores (≤7; OR: 0.95, 95% CI: 0.91-1.00).

A very similar exposure-response association was observed for low-risk deliveries with APGAR-5’ scores of 6–7 (Fig 3B) with odds increasing by 1.09 (95% CI: 1.02-1.16) with 2-day cumulative high temperature exposure (S3 Table). Again, the effect of high temperature was strongest on the day of delivery (OR: 1.09, 1.01-1.18), with no evidence of a heat effect on the preceding day (OR: 1.00, 0.92-1.07). Odds of APGAR-5’ scores 3–5 or 0–2 did not change with 2-day cumulative temperature exposure (Fig 3C and 3D).

**Fig 4:**
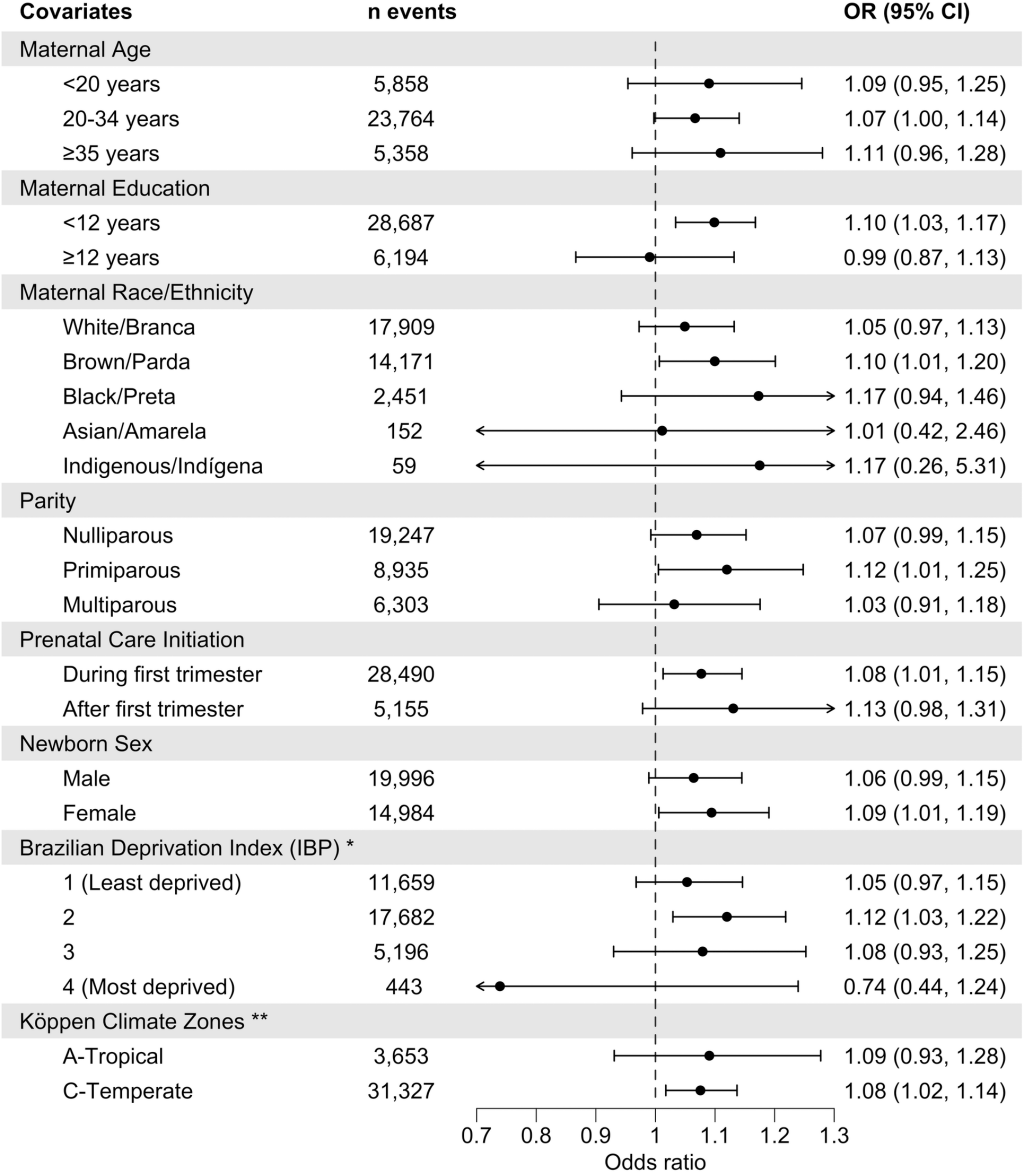
Estimated effects of high ambient temperature on odds of low APGAR-5’ score (≤7), stratified by maternal, infant, and area-level characteristics. Odds ratio (OR) and 95% CI of low APGAR-5’ score (≤7) with exposure to the 95th (versus 50th) percentile of daily mean temperature 0-1 days before delivery (2-day cumulative). The 95th and 50th percentiles (calculated from population-weighted daily mean temperatures in São Paulo state, 2013-2019) were 26.1°C and 20.9°C, respectively. * No low-risk births with low APGAR-5’ (≤7) scores occurred in municipalities in the 5th deprivation quintile. ** For Tropical zones, the 95th and 50th percentiles of population-weighted daily mean temperature (2013-2019) were 28.0°C and 23.6°C, respectively. For Temperate zones, the 95th and 50th percentiles were 25.6°C and 20.6°C, respectively.

Fig 4 presents the 2-day cumulative OR of low APGAR-5’ score (≤7) with exposure to high (95th percentile) vs moderate (50^th^ percentile) temperature, stratified by characteristics of the population. We caution that the 95% confidence intervals for many OR estimates overlap with one, and very small sample sizes among Asian/*Amarela* and Indigenous subgroups hinder interpretation. There was a tendency towards an increased odds of low APGAR-5’ score with exposure to high temperature among all subgroups, except newborns of women with ≥12 years of schooling and newborns in the most deprived municipalities (4^th^ quintile). Among the most deprived municipalities (4^th^ quintile), exposure to low temperature posed a greater risk (2-day cumulative OR: 1.57, 95% CI: 1.09-2.25; S3 Fig) than exposure to high temperature (OR: 0.74, 95% CI: 0.44-1.24).

Point estimates for the association between high temperature and low APGAR-5’ score (≤7) were similar across subgroups. We note minimal variation between strata of maternal age, newborn sex, and Köppen climate zones. However, differences were more apparent when Köppen climate zones were stratified further (S4 Table); stronger heat-low APGAR-5’ associations were seen in Tropical Rainforest (Af, OR: 1.29, 95% CI: 1.04-1.62) and Temperate Humid-subtropical zones (Cfa, 1.15, 1.04-1.27). Parity and the timing of prenatal care initiation also exhibited very little variation across strata, although associations were slightly higher in primiparous women and those with delayed prenatal care initiation. Despite overlapping error bars between strata, more notable group differences were observed for maternal education, race/ethnicity and municipality-level deprivation (IBP), with stronger evidence of adverse effects observed in women with <12 years of schooling, Brown/*Parda* women, and municipalities in the 2^nd^ deprivation quintile. Finally, across subgroups, high temperature on the day of delivery (lag 0) generally posed the greatest risk (S5 Table).

Effect estimates were robust to sensitivity analyses. Importantly, the stronger heat effect on lag 0 compared to lag 1 remained robust to any changes to model terms, lag structure, method or sample (S4 Fig). The strength of evidence for an association between high temperature and low APGAR-5’ (≤7) increased with additional flexibility in the exposure-response relationship (S6 Table, mod 4). Effect estimates were slightly lower when using the conditional quasi-Poisson model (adjusted for rate denominator, over-dispersion, and autocorrelation) (S6 Table, mod 6) and when including higher-risk births (less restricted sample; see S7 Table); however, the primary association was consistently driven by the moderately low APGAR-5’ subcategory (6–7; S7 Table).

## Discussion

This study evaluated the impact of short-term ambient heat exposure on low APGAR-5’ scores among low-risk births in São Paulo state, Brazil. High temperatures on the day of and day preceding delivery was associated with 8% increased odds of low APGAR-5’ score (≤7), primarily attributable to exposure on the day of delivery (lag 0) and driven by newborns with APGAR-5’ scores of 6–7. Elevated risks were observed among infants born to primiparous women, with fewer than 12 years of schooling, who self-declared as Brown/*Parda*, and among newborns delivered in municipalities in the second deprivation quintile.

Our findings are consistent with several previous studies conducted across diverse geographic and climatic regions, reporting an increased risk of low APGAR score with exposure to high temperature in late pregnancy. In rural Colombia, exposure to high monthly mean temperatures (≥0.7 standard deviations above the historical mean) during the third trimester was found to decrease the probability of healthy APGAR-5’ scores (≥7) by 0.3-0.5% [10]. In Australia’s Northern territory, additional hot days (daily maximum temperatures above 35°C) in the third trimester were associated with a 0.8 point reduction in overall APGAR-5’ scores [12]. However, nationally across Mexico, no effects of additional hot days (above the 90th percentile of local wet-bulb temperatures) were observed in any month during the third trimester [14]. Another study in Guangzhou, China [11], found that exposure to an additional day of extreme temperature (below 5^th^ or above 95^th^ percentiles of historical mean temperature) in the last month of pregnancy decreased summed APGAR score (measured at 1, 5 and 10 minutes post-birth) by 0.029%, however the effects of high and low temperature extremes were not reported separately, limiting comparability with our findings. Overall, the relatively coarse temporal resolution of exposure measures used in previous studies limits comparability with our findings of a same-day heat effect and may underestimate, or fail to detect, temperature effects during critical periods.

Only one previous study, based in the Ningxia Hui region of Northwest China, examined different windows of exposure during late pregnancy, including short-term effects of ambient temperature near the time of delivery. They found increased odds, between 14.0% and 47.0%, of very low APGAR-5’ scores (≤ 3) with heat exposure in different weeks and months of the third trimester [13]. Same-day (lag 0) heat exposure was not investigated, but exposure on the day before delivery (lag 1) was associated with 30.7% (7.0-59.5%) increase in odds [13]. Our analysis found no effect of high temperature on lag 1, however this discrepancy may be linked to statistical method. Tang et al. [13] appear to have used single-lag models, which do not account for correlation among exposures at adjacent lags and potentially lead to overestimation of lag-specific effects and misattribution of same-day effects to the previous day (lag 1). Alternatively, the lower and non-significant effect estimate in our study may be explained by differences in climate zones. For instance, greater risks of heat-related preterm birth have been found in arid-desert-cold climates (characteristic of Ningxia Hui) compared to humid-subtropical climates (characteristic of São Paulo) [39]. This suggests that populations in São Paulo might be better physiologically acclimatised or culturally adapted to heat than those in Ningxia Hui.

Another consideration is that our restricted ‘low-risk’ sample introduced selection bias, potentially underestimating the effects of heat by excluding the most vulnerable newborns. However, this is unlikely as the inclusion of higher-risk births in our sensitivity analysis actually yielded a smaller observed effect of ambient heat on newborns’ immediate health. Higher-risk births are typically admitted earlier and managed more intensively in specialised or tertiary care facilities equipped with air conditioning, where immediate interventions may mitigate the effects of ambient heat exposure. This may also explain the null association observed for newborns with APGAR-5’ scores of 0–5. Although we restricted our sample based on gestational age, birthweight, congenital anomalies and birth presentation, certain unobservable delivery risks – such as those related to maternal conditions or comorbidities – could not be excluded. Alternatively, the higher inherent baseline risks among these births might dilute the relative association with heat exposure. Notwithstanding, further research is needed to assess any mitigating effects of air conditioning on low APGAR-5’ score.

Importantly, the greater vulnerability of newborns with moderately low APGAR-5’ scores has significant clinical and public health implications. A cohort study in Sweden found that risk of neonatal infections, hypoglycemia, asphyxia-related complications and respiratory distress in the first 27 days of life increased with decreasing APGAR-5’ scores (9–7) in term, singleton newborns without congenital anomalies [40]. Additionally, the 6–7 category, constituting 74.3% of the newborns with low APGAR-5’ in our sample, represents a large group to which these risks are attributable.

Our subgroup analysis supports previous heat-related perinatal health research in Brazil, which indicate inequities in the vulnerability to environmental stressors [23,24]. Social determinants of health, like poverty and structural racism, may influence the exacerbating factors highlighted in the pathway between heat and low APGAR-5’ (Fig 1). Heat exposure is likely higher among lower income, less educated groups who may have more poorly insulated housing and lack air conditioning [41]. Lower socio-economic position is associated with limited healthcare access [28], which may reduce opportunities for heat-related health promotion and/or delay diagnosis/treatment for obstetric conditions among groups with already reduced baseline health [42,43]. However, we find only suggestive evidence of increased risks due to limited healthcare access, using delayed initiation of prenatal care as a proxy. Lower quality of care may also explain the observed association. Indeed, reduced referrals to specialised hospitals have previously been reported for people with lower education and from racially minoritised Black/*Preta* and Brown/*Parda* groups [29,44]. Further research into the causes of differential vulnerability and targeted health promotion efforts are critical for maternal and newborn health equity.

Although there is a suggestion of a slight protective effect of low temperature on APGAR-5’ scores, as indicated in Colombia [10], the overall association was ultimately non-significant, consistent with studies in Northwest China and Australia’s Northern territory [12,13]. However, low temperature was associated with an increased risk of low APGAR-5’ among newborns living in the most deprived municipalities, suggesting that exposure to cooler temperatures (as with warmer temperatures) interacts with social determinants to influence newborns’ immediate health. While not the primary focus of this study, due to the different biological mechanisms through which high and low temperatures may impact APGAR-5’ scores, we urge further investigation into differential cold vulnerability in this region.

Our study has several strengths. We conducted a hypothesis-driven study informed by research on pathways through which – and critical periods during which – heat exposure might impact low APGAR-5’ score. The case-crossover design was developed to study rare events caused by acute exposures [45] and findings were verified using extensive sensitivity analysis. Further research is needed to validate results in other Brazilian regions and contexts. However, the extensive coverage of SINASC data in São Paulo (99% of registered births) [46] and the minimal proportion (<0.5%) of births excluded due to missing APGAR-5’ scores, allow generalisation to low-risk births in Brazil’s most populous state [25].

We also acknowledge some key limitations. First, the case-crossover design assumes a constant rate of baseline outcome occurrence when not accounting for the exposure of interest [47]. However, high temperature has been found to increase the birth-rate on the day of, and in the days following, exposure [48]. This increase in births expands the at-risk population and, consequently, the number of deliveries with low APGAR-5’ scores. The estimated OR may thus capture the joint effect of temperature on both inducing labour (resulting in a low-risk birth) and lowering the newborn’s APGAR-5’ score. However, our sensitivity analysis using conditional quasi-Poisson regression – which is equivalent to the case-crossover design with adjustment for varying rate denominators [34] – also revealed increased risk of low APGAR-5’ (≤7) associated with high temperature on the day of delivery. This finding supports the robustness of our primary results.

Second, temperature exposures were constructed and assigned at municipality-level, which may not capture finer spatial variability, especially within larger municipalities where urbanisation and environmental conditions can vary substantially. Nonetheless, day-to-day temperature fluctuations are not highly localised. We therefore expect that short-term fluctuations were adequately captured, even if absolute temperature values varied within a municipality. Some inconsistencies between observed (weather station) and ERA5-Land reanalysis data have been reported across Brazil, however daily temperature values from both sources are very strongly correlated (*r* = 0.95) in São Paulo [49]. Unavoidable exposure misclassification might arise from the lack of data on air conditioning in both homes and hospitals. The vast proportion of births occur in health facilities, which typically have central air conditioning systems [27,50], however we were unable to ascertain the presence of air conditioning in labour wards. We could not adjust for day-specific factors that may influence neonatal outcomes, including maternal activity (e.g., travel, physical exertion) and timing of hospital admission. These factors may vary with temperature and influence APGAR-5’ scores independently, potentially biasing the estimated effect of temperature in either direction; for example, by amplifying heat-related stress or, conversely, through adaptive behaviours that reduce risk.

Finally, caution is needed in interpreting the APGAR score as the predictive value of traditionally utilised cut-offs (<7 and <4) for long-term health outcomes has been challenged [51]. However, even moderately low scores (e.g., 7) are associated with increased risks of morbidity and mortality in the neonatal period [40]. Herein, the outcome served to understand how ambient heat exposure during labour and delivery impacts the immediate health of the newborn. We defined low APGAR-5’ scores using both subcategories (0–2, 3–5, 6–7) and a threshold (≤7) aligned with Brazilian reporting conventions. Of note, APGAR-5’ scores were found to be less accurate for ascertaining the immediate health status of Black infants in the US, potentially due to the reliance on skin colour to ascertain oxygenation [52]. Given the potential for similar misclassification in Brazil, APGAR-5’ scores may not always be appropriate when distinguishing between risk profiles of different ethnoracial groups. However, ambient temperature was not expected to alter the proportion of scoring mistakes by skin colour, therefore the higher observed heat-related risk among Brown/*Parda* populations is feasibly indicative of greater heat vulnerability.

## Conclusions

This study makes a valuable contribution to the Brazilian and global evidence base on the effects of ambient heat exposure on neonatal health. Findings suggest that acute heat exposure in low-risk births is a cause for concern, especially in light of rising temperatures in highly urbanised, temperate regions like São Paulo state [22]. Our finding of increased heat-associated socio-economic vulnerability substantiates warnings that global warming will amplify the inequities in maternal and newborn health. Further research is needed to ascertain optimal ambient temperatures during labour and delivery and mediating bio-social pathways (e.g., delivery mode, care accessibility). However, we reinforce the urgent need for policies aimed at mitigating high temperature exposure near the time of delivery, including targeted health promotion initiatives and health infrastructure that is adapted at all levels.

## Supporting information

S1 Fig Geographic distribution of Brazilian Deprivation Index & Köppen climate zones in São Paulo state. Base map of municipality boundaries in São Paulo state, Brazil (year 2010) were accessed via the geobr R package [38], using official Instituto Brasileiro de Geografia e Estatística (IBGE) shapefiles for Brazil’s administrative areas (https://www.ipea.gov.br/geobr/data_gpkg/municipality/2010/35municipality_2010_simplified.gpkg). Original IBGE data are public domain and free to use, as per IBGE’s general access policy: https://www.ibge.gov.br/acesso-a-informacao.html. The geobr package is MIT-licensed and compatible with CC-BY 4.0.
(PNG)

S2 Fig Flowchart of study inclusion and exclusion criteria. (TIFF)

S1 Table Total number of low-risk births by APGAR-5’ subcategory and year in São Paulo state (2013–2019). (DOCX)

S2 Table Regression coefficients and fit statistics for models testing the association between temperature and low APGAR-5’ subcategories. Regression coefficients (Coef) are provided with their standard errors (SE), odds ratio (OR) and 95% confidence intervals (95% CI), p-values, and fit statistics. cb refers to the crossbasis exposure-lag matrix. RH refers to relative humidity and was adjusted for as a potential time-varying confounder. *Akaike Information Criterion. **Likelihood Ratio Test.
(DOCX)

S3 Table Association between high daily mean temperature and low-risk birth with low APGAR-5’ score, by subcategory. Odds ratio (OR) and 95% CI of low APGAR-5’ score subcategories (≤7, 6–7, 3–5, 0–2) with exposure to high temperature (95^th^ percentile, 26.1°C) relative to moderate temperature (50^th^ percentile, 20.9°C) 0–1 days before delivery (lags 0–1; 2-day cumulative), on the day of delivery (lag 0), and the day before delivery (lag 1). Temperature percentiles were calculated from population-weighted daily mean temperature in São Paulo state (2013–2019).
(DOCX)

S3 Fig Estimated effects of low ambient temperature on odds of low APGAR-5’ score (≤7), stratified by maternal, infant and area-level characteristics. Odds ratio (OR) and 95% CI of low APGAR score (≤7) with exposure to the 5th (versus 50th) percentile of daily mean temperature 0–1 days before delivery (2-day cumulative). The 5th and 50th percentiles (calculated from population-weighted daily mean temperature in São Paulo state, 2013–2019) were 14.8°C and 20.9°C, respectively. * No low-risk births with low APGAR-5’ score (≤7) occurred in municipalities in the 5th deprivation quintile. ** For Tropical zones, the 5th and 50th percentiles of population-weighted daily mean temperature (2013-2019) were 17.4°C and 23.6°C, respectively. For Temperate zones, the 5th and 50th percentiles were 14.6°C and 20.6°C, respectively.
(PNG)

S4 Table Association between high temperature and low APGAR-5’ (≤7), stratified by subcategories of Köppen climate zone. Odds ratio (OR) and 95% CI of low APGAR-5’ score (≤7) with exposure to high daily mean temperature (95th percentile), relative to moderate temperatures (50th percentile), 0–1 days before delivery (lags 0–1; 2-day cumulative), on the day of delivery (lag 0), and the day before delivery (lag 1). For Tropical zones, the 50th percentile was 23.6°C and the 95th percentile was 28°C. For Temperate zones, the 50th percentile was 20.6°C and the 95th percentile was 25.6°C.
(DOCX)

S5 Table Association between high temperature and low APGAR-5’ (≤7) score, stratified by individual and area-level characteristics. Odds ratio (OR) and 95% CI of low APGAR-5’ score (≤7) with exposure to high daily mean temperature (95th percentile, 26.1°C), relative to moderate temperatures (50th percentile, 20.9°C) on the day of delivery (lag 0) and the day before delivery (lag 1). * No low-risk births with low APGAR-5’ (≤7) occurred in municipalities in the 5th deprivation quintile. ** For Tropical zones, the population-weighted 95th and 50th percentiles were 28.0°C and 23.6°C, respectively. For Temperate zones, the 95th and 50th percentiles were 25.6°C and 20.6°C, respectively.
(DOCX)

S4 Fig Sensitivity analyses: Association between high temperature (lags 0 and 1) and low APGAR-5’ score (≤7). Odds ratio (OR) of low APGAR-5’ score (≤7) with exposure to high versus moderate (95^th^ vs 50^th^ percentile, 26.1°C vs 20.9°C) daily mean temperatures on the day of delivery (lag 0) and day before delivery (lag 1) for models tested in sensitivity analyses. Percentiles were calculated from population-weighted daily mean temperature in São Paulo state (2013–2019). All models, unless specified otherwise, were performed on a restricted dataset of low-risk births. Models are named following the convention: Lag structure – Crossbasis – Humidity adjustment. Crossbasis (CB) denotes the model terms used in the temperature dimension, then in the lag dimension. “lin” refers to linear. “ns” refers to a natural cubic spline, followed by the number of internal knots. The same convention applies to humidity. Humidity was always averaged (mean) over the included lag period. Where humidity was adjusted for, it is done so using a natural cubic spline with 2 knots. ‘Hnone’ refers to no humidity adjustment. For example, ‘Lag0-6 – CBns1-ns1 – Hns2’ refers to a regression analysis that modelled temperature exposures over 0-6 day lag, using a natural cubic spline with 1 internal knot in both temperature and lag dimensions, and adjusted for relative humidity using a natural cubic spline with 2 knots. We conducted the quasi-Poisson analysis excluding all-zero strata (i.e., matched day-month-year-municipality sets without any low APGAR-5’ cases). Autocorrelation was adjusted for at lags 11 and 14. *Performed on a larger sample of all singleton births between 2013–2019 in São Paulo state.
(PNG)

S6 Table Sensitivity analyses: Association between high temperature (lags 0 and 1) and low APGAR-5’ score (≤7). Odds ratio (OR) of low APGAR-5’ score (≤7) with exposure to high versus moderate (95^th^ vs 50^th^ percentile, 26.1°C vs 20.9°C) daily mean temperatures, 0–1 days before delivery (lags 0–1; 2-day cumulative), on the day of delivery (lag 0), and the day before delivery (lag 1) for models tested in sensitivity analyses. Percentiles were calculated from population-weighted daily mean temperature in São Paulo state (2013–2019). All models, unless specified otherwise, were performed on a restricted dataset of low-risk births. Models are named following the convention: Lag structure – Crossbasis – Humidity adjustment. Crossbasis (CB) denotes the model terms used in the temperature dimension, then in the lag dimension. “lin” refers to linear. “ns” refers to a natural cubic spline, followed by the number of internal knots. The same convention applies to humidity. Humidity was always averaged (mean) over the included lag period. Where humidity was adjusted for, it is done so with using a natural cubic spline with 2 knots. ‘Hnone’ refers to no humidity adjustment. For example, ‘Lag0-6 – CBns1-ns1 – Hns2’ refers to a regression analysis that modelled temperature exposures over 0–6 day lag, using a natural cubic spline with 1 internal knot in both temperature and lag dimensions, and adjusted for relative humidity using a natural cubic spline with 2 knots. We conducted the quasi-Poisson analysis excluding all-zero strata (i.e., matched day-month-year-municipality sets without any low APGAR cases). Autocorrelation was adjusted for at lags 11 and 14.
(DOCX)

S7 Table Sensitivity analysis: Association between high temperature and low APGAR-5’ in a less restricted sample. Odds ratio (OR) of low APGAR-5’ score (≤7) with exposure to high versus moderate (95^th^ vs 50^th^ percentile, 26.1°C vs 20.9°C) daily mean temperatures, 0–1 days before delivery (lags 0–1; 2-day cumulative), on the day of delivery (lag 0), and the day before delivery (lag 1). Temperature percentiles were calculated from population-weighted daily mean temperature. The sample included all singleton births between 2013–2019 in São Paulo state.
(DOCX)
