# Supplementary figures and images for: Short-term ambient heat exposure and low APGAR score in newborns: A time-stratified case-crossover analysis in São Paulo state, Brazil (2013–2019)
Source: PLOS Glob Public Health. 2025 Sep 5;5(9):e0004557. doi: 10.1371/journal.pgph.0004557 (PMC12412926; doi:10.1371/journal.pgph.0004557)

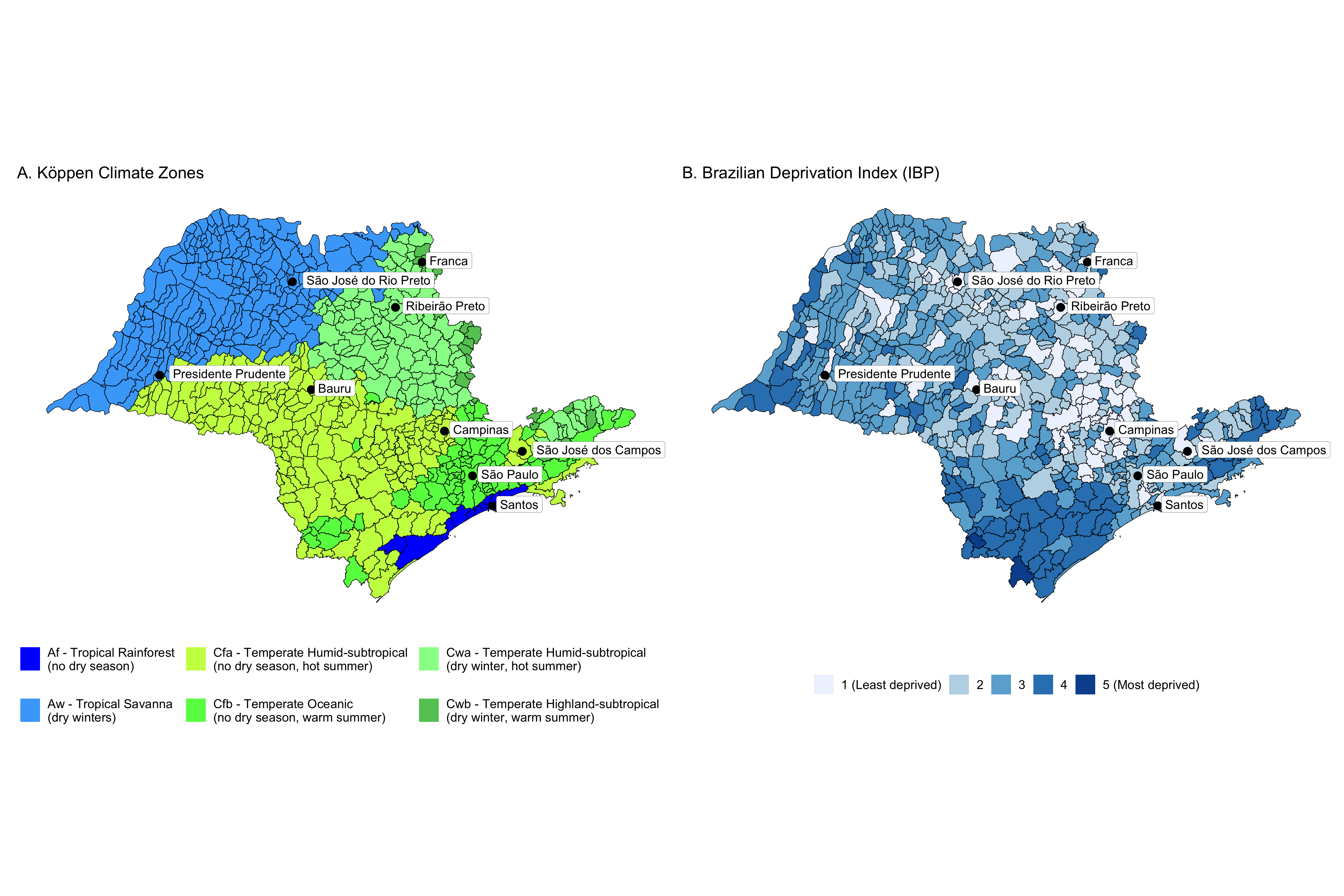

Supplement: S1 Fig — Base map of municipality boundaries in São Paulo state, Brazil (year 2010) were accessed via the geobr R package [38], using official Instituto Brasileiro de Geografia e Estatística (IBGE) shapefiles for Brazil’s administrative areas (https://www.ipea.gov.br/geobr/data_gpkg/municipality/2010/35municipality_2010_simplified.gpkg). Original IBGE data are public domain and free to use, as per IBGE’s general access policy: https://www.ibge.gov.br/acesso-a-informacao.html. The geobr package is MIT-licensed and compatible with CC-BY 4.0. (PNG) [file pgph.0004557.s001.png]

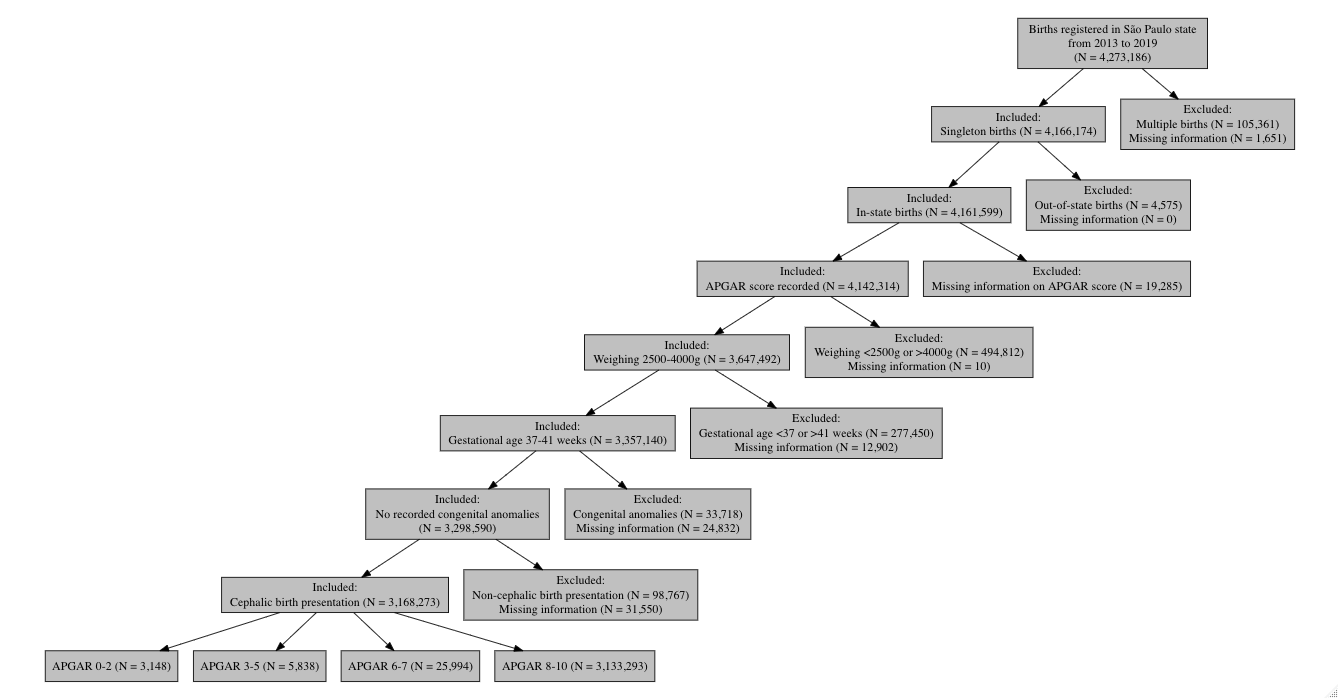

Supplement: S2 Fig — (TIFF) [file pgph.0004557.s002.tiff]

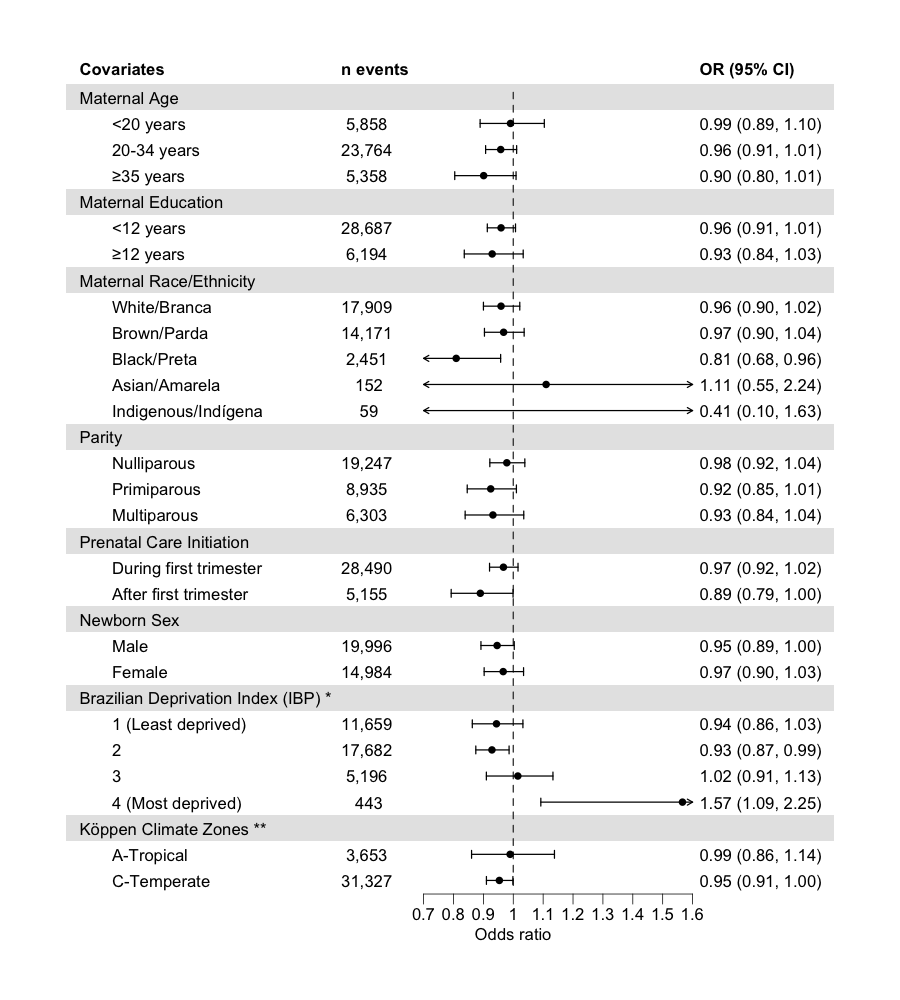

Supplement: S3 Fig — Odds ratio (OR) and 95% CI of low APGAR score (≤7) with exposure to the 5th (versus 50th) percentile of daily mean temperature 0–1 days before delivery (2-day cumulative). The 5th and 50th percentiles (calculated from population-weighted daily mean temperature in São Paulo state, 2013–2019) were 14.8°C and 20.9°C, respectively. * No low-risk births with low APGAR-5’ score (≤7) occurred in municipalities in the 5th deprivation quintile. ** For Tropical zones, the 5th and 50th percentiles of population-weighted daily mean temperature (2013-2019) were 17.4°C and 23.6°C, respectively. For Temperate zones, the 5th and 50th percentiles were 14.6°C and 20.6°C, respectively. (PNG) [file pgph.0004557.s006.png]

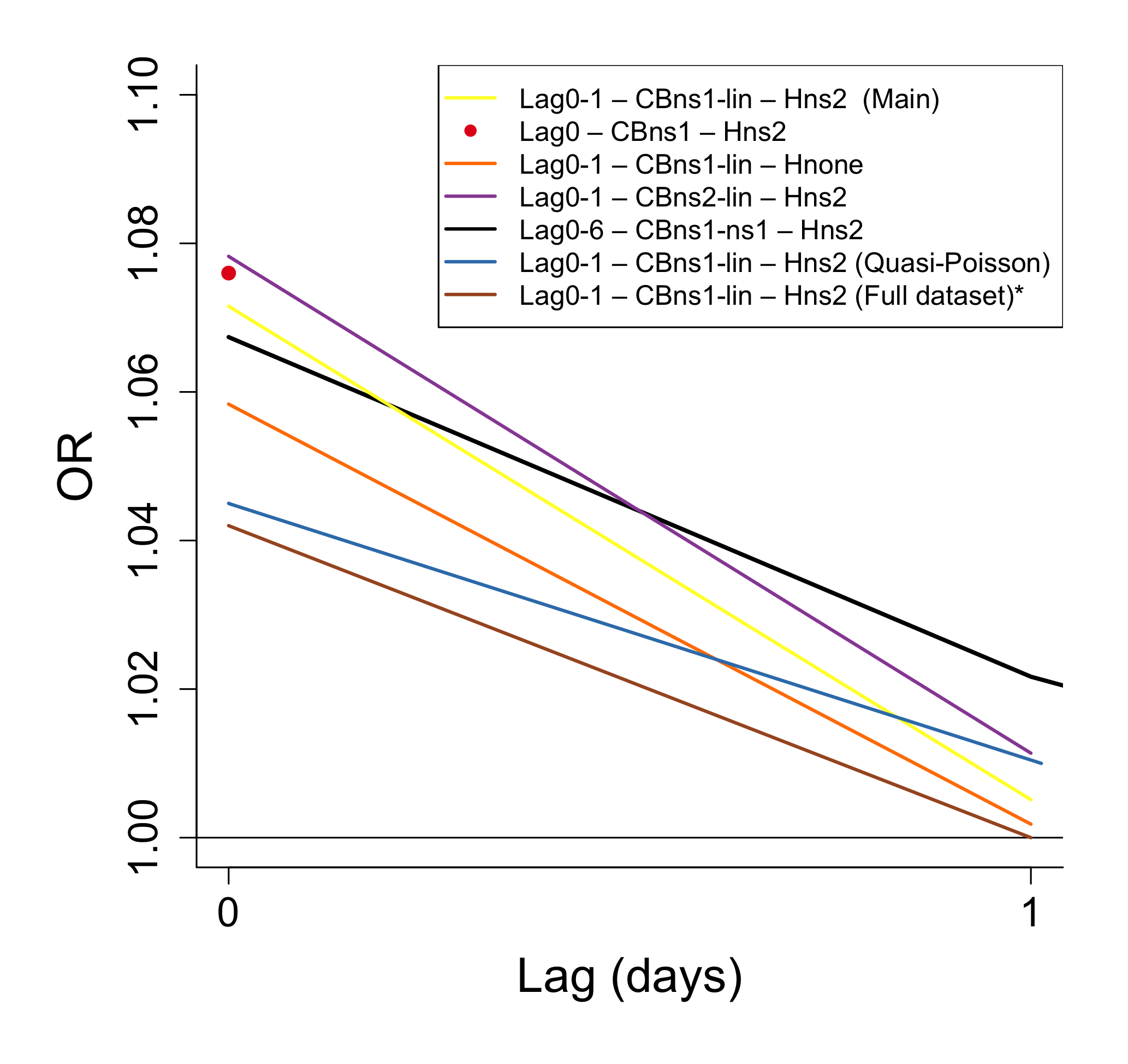

Supplement: S4 Fig — Odds ratio (OR) of low APGAR-5’ score (≤7) with exposure to high versus moderate (95th vs 50th percentile, 26.1°C vs 20.9°C) daily mean temperatures on the day of delivery (lag 0) and day before delivery (lag 1) for models tested in sensitivity analyses. Percentiles were calculated from population-weighted daily mean temperature in São Paulo state (2013–2019). All models, unless specified otherwise, were performed on a restricted dataset of low-risk births. Models are named following the convention: Lag structure – Crossbasis – Humidity adjustment. Crossbasis (CB) denotes the model terms used in the temperature dimension, then in the lag dimension. “lin” refers to linear. “ns” refers to a natural cubic spline, followed by the number of internal knots. The same convention applies to humidity. Humidity was always averaged (mean) over the included lag period. Where humidity was adjusted for, it is done so using a natural cubic spline with 2 knots. ‘Hnone’ refers to no humidity adjustment. For example, ‘Lag0-6 – CBns1-ns1 – Hns2’ refers to a regression analysis that modelled temperature exposures over 0-6 day lag, using a natural cubic spline with 1 internal knot in both temperature and lag dimensions, and adjusted for relative humidity using a natural cubic spline with 2 knots. We conducted the quasi-Poisson analysis excluding all-zero strata (i.e., matched day-month-year-municipality sets without any low APGAR-5’ cases). Autocorrelation was adjusted for at lags 11 and 14. *Performed on a larger sample of all singleton births between 2013–2019 in São Paulo state. (PNG) [file pgph.0004557.s009.png]
