# Supplementary material for: Short-term ambient heat exposure and low APGAR score in newborns: A time-stratified case-crossover analysis in São Paulo state, Brazil (2013–2019)
Source: PLOS Glob Public Health. 2025 Sep 5;5(9):e0004557. doi: 10.1371/journal.pgph.0004557 (PMC12412926; doi:10.1371/journal.pgph.0004557)
Supplement: S1 Table — (DOCX) [file pgph.0004557.s003.docx]

| **Year** | **APGAR-5’ subcategory** | | | | **Total** | **% births with APGAR-5’ ≤7** |
| --- | --- | --- | --- | --- | --- | --- |
|  | **0-2** | **3-5** | **6-7** | **8-10** |  |  |
| **2013** | 436 | 805 | 3,432 | 429,573 | 434,246 | 1.08 |
| **2014** | 493 | 865 | 3,839 | 453,080 | 458,277 | 1.13 |
| **2015** | 477 | 922 | 4,226 | 463,019 | 468,644 | 1.20 |
| **2016** | 475 | 865 | 3,896 | 446,555 | 451,791 | 1.16 |
| **2017** | 449 | 798 | 3,685 | 455,649 | 460,581 | 1.07 |
| **2018** | 404 | 807 | 3,620 | 449,850 | 454,681 | 1.06 |
| **2019** | 414 | 776 | 3,296 | 435,567 | 440,053 | 1.02 |
| **Total** | 3,148 | 5,838 | 25,994 | 3,133,293 | 3,168,273 | 1.10 |
