# Supplementary material for: Short-term ambient heat exposure and low APGAR score in newborns: A time-stratified case-crossover analysis in São Paulo state, Brazil (2013–2019)
Source: PLOS Glob Public Health. 2025 Sep 5;5(9):e0004557. doi: 10.1371/journal.pgph.0004557 (PMC12412926; doi:10.1371/journal.pgph.0004557)
Supplement: S2 Table — Regression coefficients (Coef) are provided with their standard errors (SE), odds ratio (OR) and 95% confidence intervals (95% CI), p-values, and fit statistics. cb refers to the crossbasis exposure-lag matrix. RH refers to relative humidity and was adjusted for as a potential time-varying confounder. *Akaike Information Criterion. **Likelihood Ratio Test. (DOCX) [file pgph.0004557.s004.docx]

| **Variable /Model** | **APGAR-5’ ≤ 7**  **n=34,980** | | | **APGAR-5’ 6-7**  **n= 25,994** | | | **APGAR-5’ 3-5**  **n=5,838** | | | **APGAR-5’ 0-2**  **n=3,148** | | |
| --- | --- | --- | --- | --- | --- | --- | --- | --- | --- | --- | --- | --- |
|  | **Coef**  **(SE)** | **OR**  **(95% CI)** | ***P*-value** | **Coef**  **(SE)** | **OR**  **(95% CI)** | ***P*-value** | **Coef**  **(SE)** | **OR**  **(95% CI)** | ***P*-value** | **Coef**  **(SE)** | **OR**  **(95% CI)** | ***P*-value** |
| **cb(v1.l1)** | 0.03  (0.18) | 1.03  (0.73-1.45) | 0.87 | 0.12  (0.21) | 1.13  (0.76-1.69) | 0.54 | 0.13  (0.42) | 1.14  (0.50-2.60) | 0.76 | -0.78  (0.52) | 0.46  (0.16-1.27) | 0.13 |
| **cb(v1.l2)** | 0.14  (0.32) | 1.15  (0.61-2.17) | 0.66 | -0.06  (0.38) | 0.94  (0.45-1.97) | 0.88 | 0.04  (0.78) | 1.04  (0.23-4.77) | 0.96 | 1.70  (0.95) | 5.48  (0.84-35.51) | 0.07 |
| **cb(v2.l1)** | 0.23  (0.11) | 1.25  (1.00-1.57) | **0.05** | 0.30  (0.13) | 1.35  (1.04-1.75) | **0.02** | -0.08  (0.28) | 0.92  (0.54-1.59) | 0.77 | 0.13  (0.36) | 1.14  (0.56-2.31) | 0.72 |
| **cb(v2.l2)** | -0.19  (0.20) | 0.83  (0.56-1.23) | 0.34 | -0.31  (0.23) | 0.74  (0.47-1.16) | 0.19 | 0.27  (0.50) | 1.31  (0.50-3.48) | 0.58 | -0.05  (0.65) | 0.95  (0.27-3.39) | 0.94 |
| **RH(1)** | 0.09  (0.07) | 1.10  (0.96-1.25) | 0.18 | 0.09  (0.08) | 1.10  (0.94-1.28) | 0.23 | 0.14  (0.17) | 1.15  (0.83-1.60) | 0.41 | 0.03  (0.21) | 1.03  (0.68-1.56) | 0.88 |
| **RH(2)** | 0.41  (0.24) | 1.50  (0.94-2.42) | 0.09 | 0.46  (0.28) | 1.58  (0.91-2.73) | 0.10 | 0.90  (0.60) | 2.46  (0.75-8.06) | 0.14 | -0.73  (0.74) | 0.48  (0.11-2.03) | 0.32 |
| **RH(3)** | 0.11  (0.08) | 1.12  (0.96-1.30) | 0.15 | 0.13  (0.09) | 1.14  (0.95-1.35) | 0.15 | 0.19  (0.19) | 1.21  (0.84-1.75) | 0.31 | -0.14  (0.24) | 0.87  (0.54-1.39) | 0.55 |
| **AIC*** | 103149.5 | | | 76653.85 | | | 17247.84 | | | 9263.737 | | |
| **LRT**** | X^2^=13.29; df=7; p=0.07 | | | X^2^=12.85; df=7; p=0.08 | | | X^2^=4.88; df=7; p=0.7 | | | X^2^= 7.67; df=7; p=0.4 | | |
