# Supplementary material for: Short-term ambient heat exposure and low APGAR score in newborns: A time-stratified case-crossover analysis in São Paulo state, Brazil (2013–2019)
Source: PLOS Glob Public Health. 2025 Sep 5;5(9):e0004557. doi: 10.1371/journal.pgph.0004557 (PMC12412926; doi:10.1371/journal.pgph.0004557)
Supplement: S3 Table — Odds ratio (OR) and 95% CI of low APGAR-5’ score subcategories (≤7, 6–7, 3–5, 0–2) with exposure to high temperature (95th percentile, 26.1°C) relative to moderate temperature (50th percentile, 20.9°C) 0–1 days before delivery (lags 0–1; 2-day cumulative), on the day of delivery (lag 0), and the day before delivery (lag 1). Temperature percentiles were calculated from population-weighted daily mean temperature in São Paulo state (2013–2019). (DOCX) [file pgph.0004557.s005.docx]

| **APGAR-5’ subcategory** | **Lags 0-1**  **OR (95% CI)** | **Lag 0**  **OR (95% CI)** | **Lag 1**  **OR (95% CI)** | **n events** |
| --- | --- | --- | --- | --- |
| ≤ 7 (main analysis) | **1.08 (1.02, 1.14)** | **1.07 (1.00, 1.15)** | 1.01 (0.94, 1.07) | 34,980 |
| 6-7 | **1.09 (1.02, 1.16)** | **1.09 (1.01, 1.18)** | 1.00 (0.92, 1.07) | 25,994 |
| 3-5 | 1.03 (0.90, 1.17) | 0.97 (0.82, 1.15) | 1.06 (0.90, 1.24) | 5,838 |
| 0-2 | 1.07 (0.88, 1.29) | 1.08 (0.85, 1.36) | 0.99 (0.79, 1.24) | 3,148 |
