# Supplementary material for: Short-term ambient heat exposure and low APGAR score in newborns: A time-stratified case-crossover analysis in São Paulo state, Brazil (2013–2019)
Source: PLOS Glob Public Health. 2025 Sep 5;5(9):e0004557. doi: 10.1371/journal.pgph.0004557 (PMC12412926; doi:10.1371/journal.pgph.0004557)
Supplement: S4 Table — Odds ratio (OR) and 95% CI of low APGAR-5’ score (≤7) with exposure to high daily mean temperature (95th percentile), relative to moderate temperatures (50th percentile), 0–1 days before delivery (lags 0–1; 2-day cumulative), on the day of delivery (lag 0), and the day before delivery (lag 1). For Tropical zones, the 50th percentile was 23.6°C and the 95th percentile was 28°C. For Temperate zones, the 50th percentile was 20.6°C and the 95th percentile was 25.6°C. (DOCX) [file pgph.0004557.s007.docx]

| **Köppen climate zone subcategory** | **Lags 0-1**  **OR (95% CI)** | **Lag 0**  **OR (95% CI)** | **Lag 1**  **OR (95% CI)** | **n events** |
| --- | --- | --- | --- | --- |
| Af - Tropical Rainforest (no dry season) | **1.29 (1.04, 1.62)** | 1.19 (0.93, 1.51) | 1.09 (0.86, 1.39) | 2,171 |
| Aw - Tropical Savanna (dry winters) | 0.93 (0.75, 1.16) | 0.86 (0.64, 1.17) | 1.08 (0.81, 1.43) | 1,482 |
| Cfa - Temperate Humid-subtropical (no dry season, hot summer) | **1.15 (1.04, 1.27)** | 1.11 (0.97, 1.27) | 1.03 (0.91, 1.17) | 6,971 |
| Cfb - Temperate Oceanic (no dry season, warm summer) | 1.05 (0.97, 1.13) | 1.06 (0.97, 1.16) | 0.99 (0.91, 1.08) | 20,744 |
| Cwa - Temperate Humid-subtropical (dry winter, hot summer) | 1.04 (0.90, 1.19) | 1.15 (0.94, 1.40) | 0.90 (0.75, 1.08) | 3,320 |
| Cwb - Temperate Highland-subtropical (dry winter, warm summer) | 0.88 (0.48, 1.60) | 0.48 (0.20, 1.15) | 1.84 (0.82, 4.11) | 292 |
