# Supplementary material for: Short-term ambient heat exposure and low APGAR score in newborns: A time-stratified case-crossover analysis in São Paulo state, Brazil (2013–2019)
Source: PLOS Glob Public Health. 2025 Sep 5;5(9):e0004557. doi: 10.1371/journal.pgph.0004557 (PMC12412926; doi:10.1371/journal.pgph.0004557)
Supplement: S5 Table — Odds ratio (OR) and 95% CI of low APGAR-5’ score (≤7) with exposure to high daily mean temperature (95th percentile, 26.1°C), relative to moderate temperatures (50th percentile, 20.9°C) on the day of delivery (lag 0) and the day before delivery (lag 1). * No low-risk births with low APGAR-5’ (≤7) occurred in municipalities in the 5th deprivation quintile. ** For Tropical zones, the population-weighted 95th and 50th percentiles were 28.0°C and 23.6°C, respectively. For Temperate zones, the 95th and 50th percentiles were 25.6°C and 20.6°C, respectively. (DOCX) [file pgph.0004557.s008.docx]

| **Category** | **Lag 0**  **OR (95% CI)** | **Lag 1**  **OR (95% CI)** |
| --- | --- | --- |
| Maternal Age | | |
| <20 years | 1.09 (0.92, 1.29) | 1.00 (0.85, 1.17) |
| 20-34 years | **1.09 (1.00, 1.18)** | 0.98 (0.91, 1.06) |
| ≥35 years | 0.99 (0.83, 1.17) | 1.12 (0.95, 1.33) |
| Maternal Education | | |
| <12 years | **1.09 (1.01, 1.18)** | 1.01 (0.94, 1.08) |
| ≥12 years | 0.99 (0.84, 1.16) | 1.00 (0.86, 1.17) |
| Maternal Race/Ethnicity | | |
| White/*Branca* | 1.03 (0.93, 1.13) | 1.02 (0.93, 1.12) |
| Mixed/*Parda* | **1.15 (1.03, 1.28)** | 0.96 (0.86, 1.06) |
| Black/*Preta* | 1.04 (0.80, 1.36) | 1.12 (0.87, 1.45) |
| Asian/*Amarela* | 0.34 (0.11, 1.03) | **3.01 (1.05, 8.61)** |
| Indigenous/*Indígena* | 6.69 (0.82, 54.75) | 0.18 (0.02, 1.59) |
| Parity | | |
| Nulliparous | 1.07 (0.98, 1.17) | 1.00 (0.91, 1.09) |
| Primiparous | 1.09 (0.96, 1.25) | 1.02 (0.90, 1.17) |
| Multiparous | 1.05 (0.90, 1.23) | 0.98 (0.84, 1.15) |
| Prenatal Care Initiation | | |
| During first trimester | 1.05 (0.97, 1.13) | 1.03 (0.95, 1.10) |
| After first trimester | **1.24 (1.04, 1.49)** | 0.91 (0.77, 1.08) |
| Newborn Sex | | |
| Male | 1.04 (0.95, 1.14) | 1.02 (0.94, 1.12) |
| Female | **1.12 (1.00, 1.24)** | 0.98 (0.89, 1.08) |
| Brazilian Deprivation Index (IBP) * | | |
| 1 (Least deprived) | 1.07 (0.96, 1.20) | 0.98 (0.88, 1.09) |
| 2 | 1.07 (0.97, 1.18) | 1.05 (0.95, 1.15) |
| 3 | 1.11 (0.93, 1.34) | 0.97 (0.81, 1.15) |
| 4 (Most deprived) | 0.98 (0.51, 1.87) | 0.76 (0.39, 1.48) |
| Köppen Climate Zones ** | | |
| A-Tropical | 1.03 (0.85, 1.24) | 1.06 (0.88, 1.28) |
| C-Temperate | **1.08 (1.01, 1.16)** | 1.00 (0.93, 1.06) |
