# Supplementary material for: Short-term ambient heat exposure and low APGAR score in newborns: A time-stratified case-crossover analysis in São Paulo state, Brazil (2013–2019)
Source: PLOS Glob Public Health. 2025 Sep 5;5(9):e0004557. doi: 10.1371/journal.pgph.0004557 (PMC12412926; doi:10.1371/journal.pgph.0004557)
Supplement: S6 Table — Odds ratio (OR) of low APGAR-5’ score (≤7) with exposure to high versus moderate (95th vs 50th percentile, 26.1°C vs 20.9°C) daily mean temperatures, 0–1 days before delivery (lags 0–1; 2-day cumulative), on the day of delivery (lag 0), and the day before delivery (lag 1) for models tested in sensitivity analyses. Percentiles were calculated from population-weighted daily mean temperature in São Paulo state (2013–2019). All models, unless specified otherwise, were performed on a restricted dataset of low-risk births. Models are named following the convention: Lag structure – Crossbasis – Humidity adjustment. Crossbasis (CB) denotes the model terms used in the temperature dimension, then in the lag dimension. “lin” refers to linear. “ns” refers to a natural cubic spline, followed by the number of internal knots. The same convention applies to humidity. Humidity was always averaged (mean) over the included lag period. Where humidity was adjusted for, it is done so with using a natural cubic spline with 2 knots. ‘Hnone’ refers to no humidity adjustment. For example, ‘Lag0-6 – CBns1-ns1 – Hns2’ refers to a regression analysis that modelled temperature exposures over 0–6 day lag, using a natural cubic spline with 1 internal knot in both temperature and lag dimensions, and adjusted for relative humidity using a natural cubic spline with 2 knots. We conducted the quasi-Poisson analysis excluding all-zero strata (i.e., matched day-month-year-municipality sets without any low APGAR cases). Autocorrelation was adjusted for at lags 11 and 14. (DOCX) [file pgph.0004557.s010.docx]

| **No.** | **Model description** | **Lags 0-1**  **OR (95% CI)** | **Lag 0**  **OR (95% CI)** | **Lag 1**  **OR (95% CI)** |
| --- | --- | --- | --- | --- |
| Conditional logistic regression | | | | |
| 1 | Lag0-1 – CBns1-lin – Hns2 (Main) | **1.08 (1.02, 1.14)** | **1.07 (1.00, 1.15)** | 1.01 (0.94, 1.07) |
| 2 | Lag0 – CBns1 – Hns2 | n/a | **1.08 (1.02, 1.13)** | n/a |
| 3 | Lag0-1 – CBns1-lin – Hnone | **1.06 (1.01, 1.11)** | 1.06 (0.99, 1.13) | 1.00 (0.94, 1.07) |
| 4 | Lag0-1 – CBns2-lin – Hns2 | **1.09 (1.03, 1.16)** | **1.08 (1.00, 1.16)** | 1.01 (0.94, 1.08) |
| 5 | Lag0-6 – CBns1-ns1 – Hns2 | **1.08 (1.00, 1.16)** | **1.07 (1.03, 1.11)** | **1.02 (1.00, 1.04)** |
| Conditional quasi-Poisson regression | | | | |
| 6 | Lag0-1 – CBns1-lin – Hns2 | **1.06 (1.00-1.12)** | 1.04 (0.98-1.12) | 1.01 (0.95-1.08) |
