# Supplementary material for: Short-term ambient heat exposure and low APGAR score in newborns: A time-stratified case-crossover analysis in São Paulo state, Brazil (2013–2019)
Source: PLOS Glob Public Health. 2025 Sep 5;5(9):e0004557. doi: 10.1371/journal.pgph.0004557 (PMC12412926; doi:10.1371/journal.pgph.0004557)
Supplement: S7 Table — Odds ratio (OR) of low APGAR-5’ score (≤7) with exposure to high versus moderate (95th vs 50th percentile, 26.1°C vs 20.9°C) daily mean temperatures, 0–1 days before delivery (lags 0–1; 2-day cumulative), on the day of delivery (lag 0), and the day before delivery (lag 1). Temperature percentiles were calculated from population-weighted daily mean temperature. The sample included all singleton births between 2013–2019 in São Paulo state. (DOCX) [file pgph.0004557.s011.docx]

| **Low APGAR-5’ score subcategory** | **Lags 0-1**  **OR (95% CI)** | **Lag 0**  **OR (95% CI)** | **Lag 1**  **OR (95% CI)** | **n events** |
| --- | --- | --- | --- | --- |
| ≤ 7 | **1.04 (1.00 -1.08)** | 1.04 (1.00-1.09) | 1.00 (0.96-1.04) | 76,185 |
| 6-7 | **1.05 (1.01-1.10)** | **1.06 (1.00-1.12)** | 1.00 (0.94-1.05) | 52,477 |
| 3-5 | 1.03 (0.95-1.12) | 1.00 (0.90-1.11) | 1.03 (0.93-1.14) | 14,723 |
| 0-2 | 0.99 (0.89-1.10) | 1.02 (0.89-1.16) | 0.98 (0.86-1.11) | 8,985 |
