# Supplementary material for: Short-term ambient heat exposure and low APGAR score in newborns: A time-stratified case-crossover analysis in São Paulo state, Brazil (2013–2019)
Source: PLOS Glob Public Health. 2025 Sep 5;5(9):e0004557. doi: 10.1371/journal.pgph.0004557 (PMC12412926; doi:10.1371/journal.pgph.0004557)
Supplement: Attachment — Submitted filename: Response to Editorial Team.docx [file pgph.0004557.s013.docx]

**Response to Editorial team**

Short-term ambient heat exposure and low APGAR score in newborns: A time-stratified case-crossover analysis in São Paulo state, Brazil (2013-2019)

Many thanks to Oliver Ian Tolentino and the editorial team for raising the following issue and allowing us the opportunity to rectify it. Please find a response and summary of revisions below.

Editorial team comment:

**1. Some material included in your submission may be copyrighted. According to PLOS’s copyright policy, authors who use figures or other material (e.g., graphics, clipart, maps) from another author or copyright holder must demonstrate or obtain permission to publish this material under the Creative Commons Attribution 4.0 International (CC BY 4.0) License used by PLOS journals. Please closely review the details of PLOS’s copyright requirements here: PLOS Licenses and Copyright. If you need to request permissions from a copyright holder, you may use PLOS's Copyright Content Permission form.**

**Please respond directly to this email or email the journal office and provide any known details concerning your material's license terms and permissions required for reuse, even if you have not yet obtained copyright permissions or are unsure of your material's copyright compatibility.**

**Potential Copyright Issues:**

**Fig S1: please (a) provide a direct link to the base layer of the map (i.e., the country or region border shape) and ensure this is also included in the figure legend; and (b) provide a link to the terms of use / license information for the base layer image or shapefile. We cannot publish proprietary or copyrighted maps (e.g. Google Maps, Mapquest) and the terms of use for your map base layer must be compatible with our CC-BY 4.0 license.**

**Note: if you created the map in a software program like R or ArcGIS, please locate and indicate the source of the basemap shapefile onto which data has been plotted.**

**If your map was obtained from a copyrighted source please amend the figure so that the base map used is from an openly available source. Alternatively, please provide explicit written permission from the copyright holder granting you the right to publish the material under our CC-BY 4.0 license.**

**Please note that the following CC BY licenses are compatible with PLOS license: CC BY 4.0, CC BY 2.0 and CC BY 3.0, meanwhile such licenses as CC BY-ND 3.0 and others are not compatible due to additional restrictions.**

**If you are unsure whether you can use a map or not, please do reach out and we will be able to help you. The following websites are good examples of where you can source open access or public domain maps:**

*** U.S. Geological Survey (USGS) - All maps are in the public domain. (http://www.usgs.gov)**

*** PlaniGlobe - All maps are published under a Creative Commons license so please cite “PlaniGlobe, http://www.planiglobe.com, CC BY 2.0” in the image credit after the caption. (http://www.planiglobe.com/?lang=enl)**

*** Natural Earth - All maps are public domain. (**[**http://www.naturalearthdata.com/about/terms-of-use/)**](http://www.naturalearthdata.com/about/terms-of-use/))

Authors’ response:

Thank you for bringing this to our attention. Figure S1 was created by us in RStudio using the ‘geobr’ package, which is developed and maintained by the Institute for Applied Economic Research (Ipea) in Brazil. The geobr package facilitates the creation of maps from official spatial shapefiles and datasets produced by the Instituto Brasileiro de Geografia e Estatistica (IBGE). Original IBGE data are public domain and free to use, as per IBGE’s access policy: <https://www.ibge.gov.br/acesso-a-informacao.html>. The geobr package is MIT-licensed and compatible with CC‑BY 4.0. Licensing information can be found at <https://cran.r-project.org/web/packages/geobr/index.html> and <https://ipeagit.github.io/geobr/> and is as follows:

Copyright (c) 2019, Ipea

Permission is hereby granted, free of charge, to any person obtaining a copy of this software and associated documentation files (the "Software"), to deal in the Software without restriction, including without limitation the rights to use, copy, modify, merge, publish, distribute, sublicense, and/or sell copies of the Software, and to permit persons to whom the Software is furnished to do so, subject to the following conditions:

The above copyright notice and this permission notice shall be included in all copies or substantial portions of the Software.

THE SOFTWARE IS PROVIDED "AS IS", WITHOUT WARRANTY OF ANY KIND, EXPRESS OR IMPLIED, INCLUDING BUT NOT LIMITED TO THE WARRANTIES OF MERCHANTABILITY, FITNESS FOR A PARTICULAR PURPOSE AND NONINFRINGEMENT. IN NO EVENT SHALL THE AUTHORS OR COPYRIGHT HOLDERS BE LIABLE FOR ANY CLAIM, DAMAGES OR OTHER LIABILITY, WHETHER IN AN ACTION OF CONTRACT, TORT OR OTHERWISE, ARISING FROM, OUT OF OR IN CONNECTION WITH THE SOFTWARE OR THE USE OR OTHER DEALINGS IN THE SOFTWARE.

In accordance with your recommendations, we have made the following changes (additional text in **bold**):

- Cited the package used to create the maps in the Methods section

*“All analyses and figures were conducted in R (4.2.3) using Rstudio and facilitated by the following packages: survival [35], dlnm [36], splines [37] and* ***geobr [38]****.”*

- Included the full citation in the References section:

“***Pereira RHM, Gonçalves C, et al. geobr: Download Official Spatial Data Sets of Brazil. R package version 1.9.1. 2024. Available from:*** [***https://github.com/ipeaGIT/geobr***](https://github.com/ipeaGIT/geobr)***,*** [***https://ipeagit.github.io/geobr/***](https://ipeagit.github.io/geobr/)***.****”*

- Edited the supporting information captions for the relevant figure (Fig S1):

*“Fig S1. Geographic distribution of Brazilian Deprivation Index & Köppen climate zones in São Paulo state.* ***Base map of municipality boundaries in São Paulo state, Brazil (year 2010) were accessed via the geobr R package [38], using official Instituto Brasileiro de Geografia e Estatística (IBGE) shapefiles for Brazil’s administrative areas (***[***https://www.ipea.gov.br/geobr/data_gpkg/municipality/2010/35municipality_2010_simplified.gpkg***](https://www.ipea.gov.br/geobr/data_gpkg/municipality/2010/35municipality_2010_simplified.gpkg)***). Original IBGE data are public domain and free to use, as per IBGE’s access policy:*** [***https://www.ibge.gov.br/acesso-a-informacao.html***](https://www.ibge.gov.br/acesso-a-informacao.html)***. The geobr package is MIT-licensed and compatible with CC‑BY 4.0****.”*

We trust that the above revisions fully comply with PLOS’ Copyright Policy, but please do not hesitate to contact us if additional information is required.

With thanks and best wishes on behalf of all co-authors,

Cherie Part
