# Supplementary material for: Short-term ambient heat exposure and low APGAR score in newborns: A time-stratified case-crossover analysis in São Paulo state, Brazil (2013–2019)
Source: PLOS Glob Public Health. 2025 Sep 5;5(9):e0004557. doi: 10.1371/journal.pgph.0004557 (PMC12412926; doi:10.1371/journal.pgph.0004557)
Supplement: Peer review history

# Peer review: Decision Letter 0

**DOI:** 10.1371/journal.pgph.0004557.r001

**Authors:** Vanessa Moraes Bezerra

4 Jun 2025

PGPH-D-25-00804



Exposição de curto prazo ao calor ambiente e baixo escore de APGAR em recém-nascidos: Uma análise de cruzamento de casos estratificada temporalmente no estado de São Paulo, Brasil (2013-2019)



PLOS Global Public Health



Prezado Dr. Part,



Obrigado por enviar seu manuscrito para a PLOS Global Public Health. Após cuidadosa análise, acreditamos que ele tem mérito, mas não atende totalmente aos critérios de publicação da PLOS Global Public Health conforme estão atualmente. Portanto, convidamos você a enviar uma versão revisada do manuscrito que aborde os pontos levantados durante o processo de revisão.



Envie seu manuscrito revisado até Jul 04 2025 11:59PM. Caso precise de mais tempo para concluir suas revisões, responda a esta mensagem ou entre em contato com o escritório do periódico pelo e-mail globalpubhealth@plos.org. Quando estiver pronto para enviar sua revisão, acesse https://www.editorialmanager.com/pgph/ e selecione a pasta "Submissões que Necessitam de Revisão" para localizar o arquivo do seu manuscrito.



Inclua os seguintes itens ao enviar seu manuscrito revisado:



Uma carta de réplica que responda a cada ponto levantado pelo editor e pelo(s) revisor(es). Você deve enviar esta carta como um arquivo separado, intitulado "Resposta aos Revisores".



Uma cópia corrigida do seu manuscrito, destacando as alterações feitas na versão original. Você deve enviá-la como um arquivo separado, denominado "Manuscrito Revisado com Controle de Alterações".



Uma versão sem marcação do seu artigo revisado, sem alterações rastreadas. Você deve enviá-la como um arquivo separado, denominado "Manuscrito".



As diretrizes para reenviar seus arquivos de figuras estão disponíveis abaixo dos comentários do revisor no final desta carta.



Aguardamos ansiosamente o recebimento do seu manuscrito revisado.



Atenciosamente,



Vanessa Moraes Bezerra



Editora Acadêmica

Requisitos



do PLOS Global Public Health Journal:

Revise sua lista de referências para garantir que esteja completa e correta. Se você citou artigos que foram retratados, inclua a justificativa para isso no texto do manuscrito ou remova essas referências e substitua-as por referências atuais relevantes. Quaisquer alterações na lista de referências devem ser mencionadas na carta de refutação que acompanha o seu manuscrito revisado. Se você precisar citar um artigo retratado, indique o status de retratado do artigo na lista de Referências e também inclua uma citação e a referência completa para o aviso de retratação.

Comentários adicionais do editor (se fornecidos):

[Observação: a marcação HTML está abaixo. Por favor, não edite.]

Comentários dos revisores:

Respostas dos revisores às perguntas

**

Comentários ao Autor

**

1. Este manuscrito atende

aos critérios de publicação

da PLOS Global Public Health ? O manuscrito é tecnicamente sólido e os dados corroboram as conclusões? O manuscrito deve descrever uma pesquisa metodológica e eticamente rigorosa, com conclusões apropriadas com base nos dados apresentados.



Avaliador nº 1: Sim



Avaliador nº 2: Sim

**********

2. A análise estatística foi realizada de forma adequada e rigorosa?



Avaliador nº 1: Sim



Avaliador nº 2: Sim

**********

3. Os autores disponibilizaram integralmente todos os dados que fundamentam as descobertas em seu manuscrito (consulte a Declaração de Disponibilidade de Dados no início do arquivo PDF do manuscrito)?



A

política de dados da PLOS

exige que os autores disponibilizem integralmente, sem restrições, todos os dados que fundamentam as descobertas descritas em seu manuscrito, com raras exceções. Os dados devem ser fornecidos como parte do manuscrito ou de suas informações complementares, ou depositados em um repositório público. Por exemplo, além das estatísticas resumidas, os pontos de dados por trás das médias, medianas e medidas de variância devem estar disponíveis. Se houver restrições ao compartilhamento público de dados — por exemplo, privacidade dos participantes ou uso de dados de terceiros —, elas devem ser especificadas.



Avaliador nº 1: Sim



Avaliador nº 2: Sim

**********

4. O manuscrito é apresentado de forma inteligível e escrito em inglês padrão?



A PLOS Global Public Health não realiza a revisão de manuscritos aceitos, portanto, a linguagem dos artigos submetidos deve ser clara, correta e inequívoca. Quaisquer erros tipográficos ou gramaticais devem ser corrigidos na revisão; portanto, anote aqui quaisquer erros específicos.



Avaliador nº 1: Sim



Avaliador nº 2: Sim

**********

5. Comentários da revisão para o autor.



Use o espaço fornecido para explicar suas respostas às perguntas acima. Você também pode incluir comentários adicionais para o autor, incluindo preocupações sobre publicação dupla, ética em pesquisa ou ética de publicação. (Envie sua revisão como anexo se ela exceder 20.000 caracteres.)



Revisor nº 1: Título do artigo: Exposição ao calor ambiente de curta duração e baixo escore de APGAR em recém-nascidos: Uma análise de caso cruzado estratificada no tempo no estado de São Paulo, Brasil (2013-2019)



Autor correspondente: Cherie Part, Ph.D.London School of Hygiene & Tropical Medicine, Londres, REINO UNIDO DA GRÃ-BRETANHA E IRLANDA DO NORTE



1. Introdução e Objetivos



Comentário: O título não esclarece que a exposição ao calor ambiental se refere às mães. Sugiro escrevê-lo. Exemplo: Exposição materna a altas temperaturas ambientes...



Objetivo do estudo: Está claramente definido, mas seria interessante se fosse mais específico, indicando que se trata da exposição materna a ambientes com altas temperaturas ambientes



2.



Comentário da Revisão da Literatura: A revisão abrange os principais estudos relacionados, citando autores clássicos e recentes. Apresenta uma base teórica adequada para o tema estudado.



3.



Comentário da Metodologia: A descrição dos métodos é adequada, com detalhes sobre o procedimento de coleta de dados, critérios de inclusão/exclusão e análise estatística utilizada.



4. Resultados:



Comentário: Os resultados da pesquisa foram descritos de forma organizada e detalhada. Os gráficos e tabelas contêm informações bem ilustradas.



5.



Discussão: A discussão interpreta bem os resultados, mas houve pouca comparação com estudos anteriores. Apenas 4 artigos foram utilizados para comparar os resultados.



Sugestão: Adicionar mais artigos para comparar os resultados.



6. Conclusão



: A conclusão resumiu bem os pontos principais, destacando as contribuições específicas do estudo e sugestões concretas para pesquisas futuras.



7. Originalidade e Contribuição



: O artigo traz uma contribuição relevante para a área, com um tema relevante e inovador.



8. Redação e Apresentação



: O texto está bem escrito, com linguagem clara e objetiva.



9. Recomendações Gerais:



Comentários: Aprimorar o título de acordo com a orientação descrita acima.



Adicionar mais artigos ao comparar os resultados encontrados.



Revisor nº 2: O tema do estudo é interessante e relevante para a saúde pública. O estudo foi bem conduzido metodologicamente e traz novas evidências à literatura sobre exposição ao calor ambiental e escores de APGAR em recém-nascidos.



Parabenizo os autores pelo rigor metodológico de seu trabalho e sugiro pequenos ajustes para aprimorar ainda mais o manuscrito.



- As análises estratificadas sugerem que as associações foram observadas apenas em recém-nascidos com escores de APGAR entre 6 e 7, classificados como “moderadamente baixo”. Nesse sentido, as implicações clínicas desses resultados podem não ser tão evidentes para os leitores. Portanto, sugiro incluir um pequeno trecho sobre os possíveis impactos desses escores na saúde perinatal na seção Discussão.



- Embora o delineamento case-crossover tenha sido ajustado para fatores de confusão que não variam ao longo do tempo, algumas variáveis específicas do dia (por exemplo, atividade materna no dia do parto, horário de admissão hospitalar) não foram consideradas. Portanto, considero importante reconhecer essa limitação na Discussão do estudo.

**********

6. Os autores da PLOS têm a opção de publicar o histórico de revisão por pares de seus artigos (

o que isso significa?

). Se publicado, isso incluirá sua revisão por pares completa e quaisquer arquivos anexados.

**

Deseja que sua identidade seja pública para esta revisão por pares?

**

Se escolher "não", sua identidade permanecerá anônima, mas sua revisão ainda poderá ser pública.



Para obter informações sobre essa opção, incluindo a revogação do consentimento, consulte nossa

Política de Privacidade

.



Avaliador nº 1: Não



Avaliador nº 2: Não

**********

[NOTA: Se os comentários dos revisores foram enviados como um arquivo anexo, eles serão anexados a este e-mail e acessíveis através do site de submissão. Faça login em sua conta, localize o registro do manuscrito e verifique o link de ação "Visualizar Anexos". Se este link não aparecer, não há arquivos anexos.]



Ao revisar sua submissão, faça upload dos arquivos das figuras para a ferramenta de diagnóstico digital Preflight Analysis and Conversion Engine (PACE), https://pacev2.apexcovantage.com/. O PACE ajuda a garantir que as figuras atendam aos requisitos do PLOS. Para usar o PACE, você deve primeiro se registrar como usuário. O registro é gratuito. Em seguida, faça login e navegue até a aba UPLOAD, onde você encontrará instruções detalhadas sobre como usar a ferramenta. Se você encontrar algum problema ou tiver alguma dúvida ao usar o PACE, envie um e-mail para o PLOS em figures@plos.org. Observe que os arquivos de Informações Complementares não precisam desta etapa.

# Author comment: Author response to Decision Letter 1

**DOI:** 10.1371/journal.pgph.0004557.r002

6 Aug 2025

Attachment Submitted filename: Response to Editorial Team.docx

# Editor report: Decision Letter 1

**DOI:** 10.1371/journal.pgph.0004557.r003

**Authors:** Vanessa Moraes Bezerra

14 Aug 2025

Short-term ambient heat exposure and low APGAR score in newborns: A time-stratified case-crossover analysis in São Paulo state, Brazil (2013-2019)

PGPH-D-25-00804R1

Dear Dr Part,

We are pleased to inform you that your manuscript 'Short-term ambient heat exposure and low APGAR score in newborns: A time-stratified case-crossover analysis in São Paulo state, Brazil (2013-2019)' has been provisionally accepted for publication in PLOS Global Public Health.

Before your manuscript can be formally accepted you will need to complete some formatting changes, which you will receive in a follow up email. A member of our team will be in touch with a set of requests.

Please note that your manuscript will not be scheduled for publication until you have made the required changes, so a swift response is appreciated.

IMPORTANT: The editorial review process is now complete. PLOS will only permit corrections to spelling, formatting or significant scientific errors from this point onwards. Requests for major changes, or any which affect the scientific understanding of your work, will cause delays to the publication date of your manuscript.

If your institution or institutions have a press office, please notify them about your upcoming paper to help maximize its impact. If they'll be preparing press materials, please inform our press team as soon as possible -- no later than 48 hours after receiving the formal acceptance. Your manuscript will remain under strict press embargo until 2 pm Eastern Time on the date of publication. For more information, please contact globalpubhealth@plos.org.

Thank you again for supporting Open Access publishing; we are looking forward to publishing your work in PLOS Global Public Health.

Best regards,

Vanessa Moraes Bezerra

Academic Editor

PLOS Global Public Health

***********************************************************

Reviewer Comments (if any, and for reference):
